# COVID-19 Awareness Among Dental Professionals in Indonesia

**DOI:** 10.3389/fmed.2020.589759

**Published:** 2020-11-04

**Authors:** Armelia Sari Widyarman, Endang W. Bachtiar, Citra Fragrantia Theodorea, M. Ihsan Rizal, M. Orliando Roeslan, Melanie S. Djamil, Didi N. Santosa, Boy M. Bachtiar

**Affiliations:** ^1^Department of Microbiology, Faculty of Dentistry, Trisakti University, Jakarta, Indonesia; ^2^Department of Oral Biology, Faculty of Dentistry, Universitas Indonesia, Depok, Indonesia; ^3^Department of Oral Biology, Faculty of Dentistry, Trisakti University, Jakarta, Indonesia

**Keywords:** SARS-COV-2, knowledge, dentist, COVID-19, awareness

## Abstract

**Background:** The COVID-19 pandemic caused by SARS-CoV-2 has claimed thousands of lives worldwide. To enhance knowledge and awareness of COVID-19, considerable online resources have been made available, including aspects related to the dental profession. The study aim was to examine the knowledge, perception, and attitude of dental professionals in Indonesia toward COVID-19. We conducted a survey via a questionnaire created using Google docs and distributed to 632 members of the Indonesian Dental Association in the context of a webinar hosted by the Indonesian Oral Biology Association on first June, 2020.

**Materials and Methods:** The questionnaire consisted of 17 items pertaining to demographic data, knowledge and virus identification, awareness regarding drugs commonly used in dentistry during pandemic and research opportunities. Participants were asked to complete the questionnaire after the webinar by choosing one answer to each question. For the analysis, participants were divided into three groups according to their professional background i.e., employment at national hospital, private hospital, or academic faculty. Data were analyzed using descriptive statistics and expressed as frequencies and percentages. The chi-square test was used to investigate the association between professional activity and the level of knowledge, perceptions, and attitudes about COVID-19.

**Results:** Sixty percent of the participants correctly identified the pathogenesis of the disease. This knowledge was not associated with their professional affiliation (*p* = 0.95). Sixty-seven percentage had comprehensive knowledge about virus detection methods. This knowledge was not associated with their affiliation either (*p* = 0.54). Questions regarding drugs of choice, prevention, and the spread of COVID-19 were correctly answered by 89, 96, and 82% of the participants, respectively. Knowledge of these aspects were significantly associated with the professional affiliation (*p* < 0.05). All respondents were optimistic regarding research opportunities (*p* < 0.01). Respondents from academics were more interested in joining COVID-19-related research projects with governmental institutions (*p* < 0.01).

**Conclusion:** Knowledge and awareness of COVID-19 among Indonesian dentists are reasonably good. However, further improvement would be beneficial to manage patients during this pandemic. As the number of COVID-19 cases continue to rise in Indonesia, it is important that dentists keep abreast of the updated knowledge on this moving field. Dentist knowledge on infection control should be strengthened through continuous educational programs.

## Introduction

Since it was first reported in Wuhan City, Hubei Province, Central China in December 2019, coronavirus disease 2019 (COVID-19) has become a major health concern worldwide (1). COVID-19 is caused by a novel coronavirus called severe acute respiratory syndrome coronavirus 2 (SARS-CoV-2) which causes pneumonia, with symptoms ranging from mild to deadly. SARS-CoV-2 infection can cause an acute inflammatory response (cytokine storm) and respiratory failure (2, 3). The World Health Organization (WHO) declared COVID-19 a pandemic on March 11, 2020 (4). As of July 6, 2020, COVID-19 had spread to 216 countries, causing 11,496,926 confirmed cases and over 535,390 deaths (5). Indonesia reported its first two cases on March 2, 2020. By July 6, it had reported 63,749 cases and 3,171 deaths (6). Indonesia currently has the highest COVID-19-related mortality rate in Southeast Asia.

Dental schools and their affiliated hospitals, as well as research laboratories have also been significantly affected by the pandemic. As a consequence, most research work has been stopped or undergone radical changes with severe limitations. These difficulties have been compounded by the absence of technical support as some experienced laboratory personnel belong to high-risk groups. However, this challenging situation has offered an opportunity to re-evaluate the our knowledge and understanding of the infection control measures related to dentistry and formulate new strategies in the post-COVID era (7).

The risk of viral transmission is higher for those who are close to or work near patients, such as relatives and health workers. To evaluate the preparedness of dentists to cope with the pandemic, many researchers around the globe are trying to assess their knowledge of COVID-19. In this study, we included the knowledge of characterization and method identification of SARS-CoV-2.

Due to the involvement of aerosol generating procedures, the dental profession is regarded as one of the occupations at the highest risk of SARS-CoV-2 infection. Therefore, it is critical that the risk of transmission through dental procedures is minimized through proper understanding and actions. Dentists should be well-aware of the characteristics of SARS-CoV-2 and new infection control standards. In the Indonesian context, many dentists, particularly in the private practice has opted to perform only emergency procedures during the pandemic. Hence, dental pain of the patients has been commonly managed with prescription of analgesics. In order to enhance knowledge on COVID-19 and the management of dental patients during the pandemic, Indonesian Oral Biology Association organized a series of webinars through Zoom platform. However, there are no studies which has assessed the knowledge, perception, and attitude of Indonesian dentists on COVID-19. Considering this research gap, in the present study we evaluated the level of knowledge on SARS-CoV-2 and COVID-19 among dental professionals in Indonesia. In this study, we highlighted the most important research questions concerning the knowledge of the virus, the pathogenesis as well as the virus detection and infection prevention of COVID-19.

## Materials and Methods

This was a survey-based study conducted via a questionnaire distributed to all dentist members of the Indonesian Dental Association attending a webinar hosted by the Indonesian Oral Biology Association on first June, 2020 on COVID-19 related topic. The questionnaire was prepared in Indonesian native language. The original and translated version the questionnaire to English is given in the Supplementary Material. Google docs were used as a platform to create the questionnaire, which was then distributed online. Participants from any region of Indonesia were granted password-protected access to a URL hosting the questionnaire. A unique study ID ensured confidentiality of all self-reported data. The participants' responses were stored in a cloud database, where the data were automatically sorted, scaled, and scored using custom Microsoft Office Excel formulas. This study was approved by the Institutional Review Board of Faculty of Dentistry, Trisakti University (Jakarta, Indonesia) with approval number: 360/s3/KEPK/FKG/2020.

The questionnaire consisted of 17 items, including four pertaining to demographic information (age, gender, and residence and work locations), three regarding knowledge about the virus, two regarding the method identification of COVID-19, two regarding knowledge about its pathogenesis, two about infection control and precautions, two pertaining to awareness regarding drugs commonly used in dentistry during the pandemic, one regarding research opportunities, and one regarding research collaborations (Questionnaire is given in the Supplementary Material). The questionnaire was developed and confirmed after review by a panel of experts (EWB, BMB, MSD, and DNS). Construct validity of this questionnaire was done through intense discussion. The reliability of the questionnaire was statistically scaled as alpha Cronbach = 0.66. The participants were asked to choose one answer to each question. The level of optimism regarding research opportunities during the pandemic was assessed with a question that had three possible answers: “optimistic,” “doubtful,” and “pessimistic.” Participants' willingness to cooperate with the government in COVID-19-related research projects was assessed with a question that had three possible answers: “yes,” “maybe,” and “no.”

For the statistical analysis, the participants were divided into three groups according to their professional affiliation i.e., dentists employed at national hospital consist of dentist who work in the health center and government hospital. Dentists employed at private hospital consist of dentist who work in private hospital, private dental practice or dental clinic and employment as an academic in a dental school. Researchers downloaded questionnaire responses in multiple formats (Microsoft Office Excel), which were then analyzed using descriptive statistics and expressed as frequencies and percentages. The response rate to the study was 100%. We exclude participants who did not answer all the questions. We analyzed the correctness rate by calculating the average percentage of correct answer for all related questions in each topic. The chi-square test was used to investigate associations between professional background and knowledge, perceptions, attitudes about COVID-19 and to compare the levels of optimism regarding research opportunities and willingness to collaborate with the government between the three groups. IBM SPSS Statistics version 25 (IBM, Armonk, NY, USA) was used for the statistical analyses. The *p* < 0.05 was considered statistically significant.

## Results

The study cohort consisted of a total of 632 dentists who participated in the survey. Demographic features include 524 (82.9%) female and 108 (17.1%) male, age range from 20 to 65 years old, and from 28 provinces in Indonesia. The distribution of the professional affiliation includes 175 (27.7%) academics, 158 (25%) national hospital practitioners, and 299 (47.3%) private hospital/private practice practitioners as shown in Table 1.

**Table 1:** Demographic characterisatic of participants.

**Characteristic**		***n***	**%**
Sex	Woman	524	82.9
	Man	108	17.1
Age	20–30 years	139	22.0
	31–40 years	188	29.7
	41–50 years	146	23.1
	51–65 years	159	25.2
Occupation	Academic	175	27.7
	Government hospital	158	25
	Private hospital/private practice	299	47.3
Province	Riau	10	1.6
	Riau Islands	7	1.1
	Bangka-Belitung Islands	5	0.8
	Aceh	6	1.0
	North Sumatra	10	1.6
	West Sumatra	12	1.9
	South Sumatera	14	2.2
	Bengkulu	3	0.5
	Jambi	2	0.3
	Lampung	3	0.5
	Banten	42	6.7
	Jakarta	155	24.6
	West Java	109	17.3
	Central Java	54	8.6
	Yogyakarta	25	4.0
	East Java	88	13.9
	Bali	18	2.9
	East Nusa Tenggara	2	0.3
	West Nusa Tenggara	4	0.6
	Central Kalimantan	4	0.6
	North Kalimantan	4	0.6
	South Kalimantan	1	0.2
	West Kalimantan	3	0.5
	East Kalimantan	10	1.6
	South Sulawesi	29	4.6
	North Sulawesi	3	0.5
	North Maluku	1	0.2
	Papua	7	1.1

**Figure 1:**
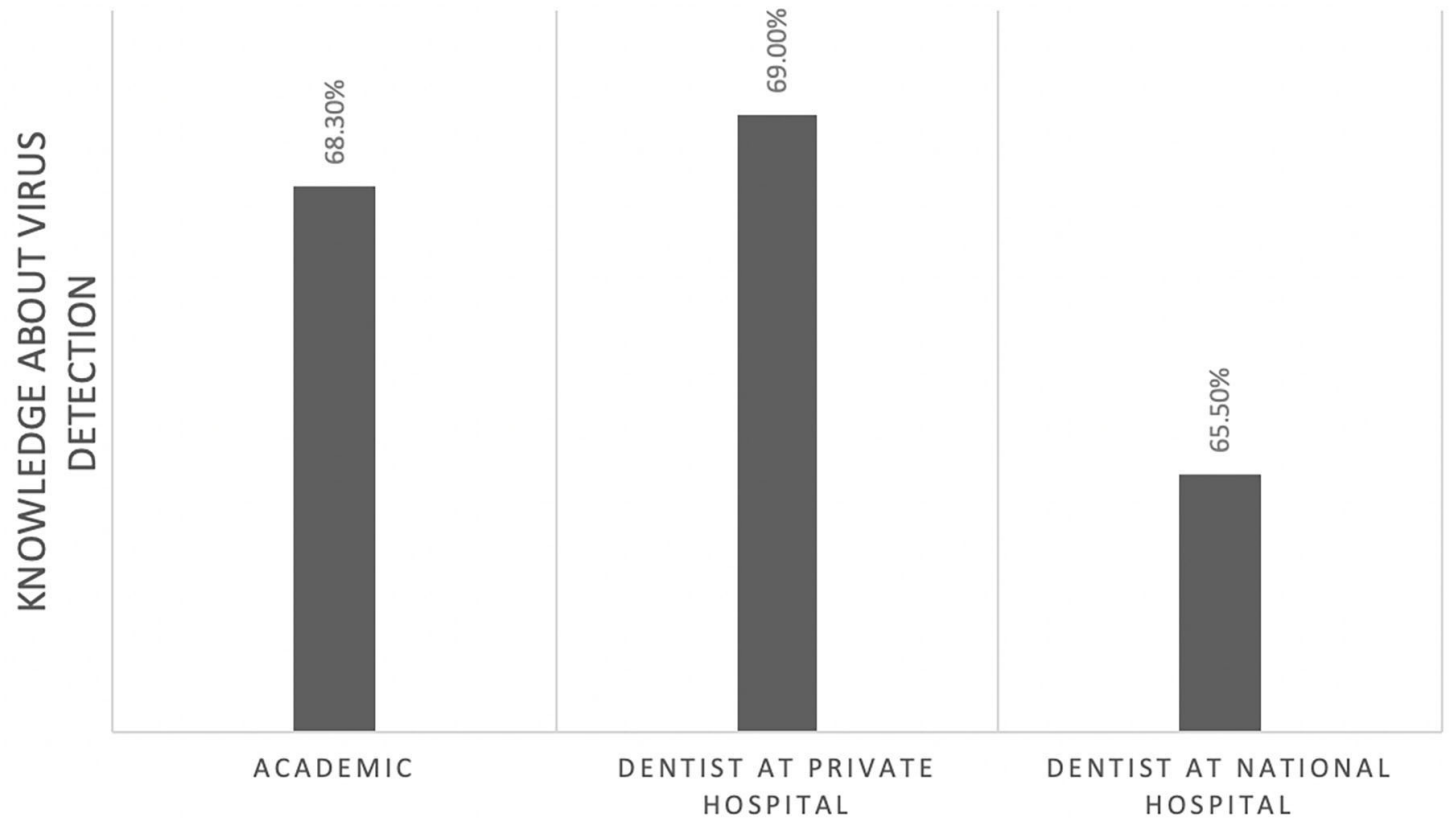
Sixty percent of the participants correctly identified the pathogenesis of COVID-19. This knowledge was not associated with the professional affiliation (*p* = 0.95).

**Figure 2:**
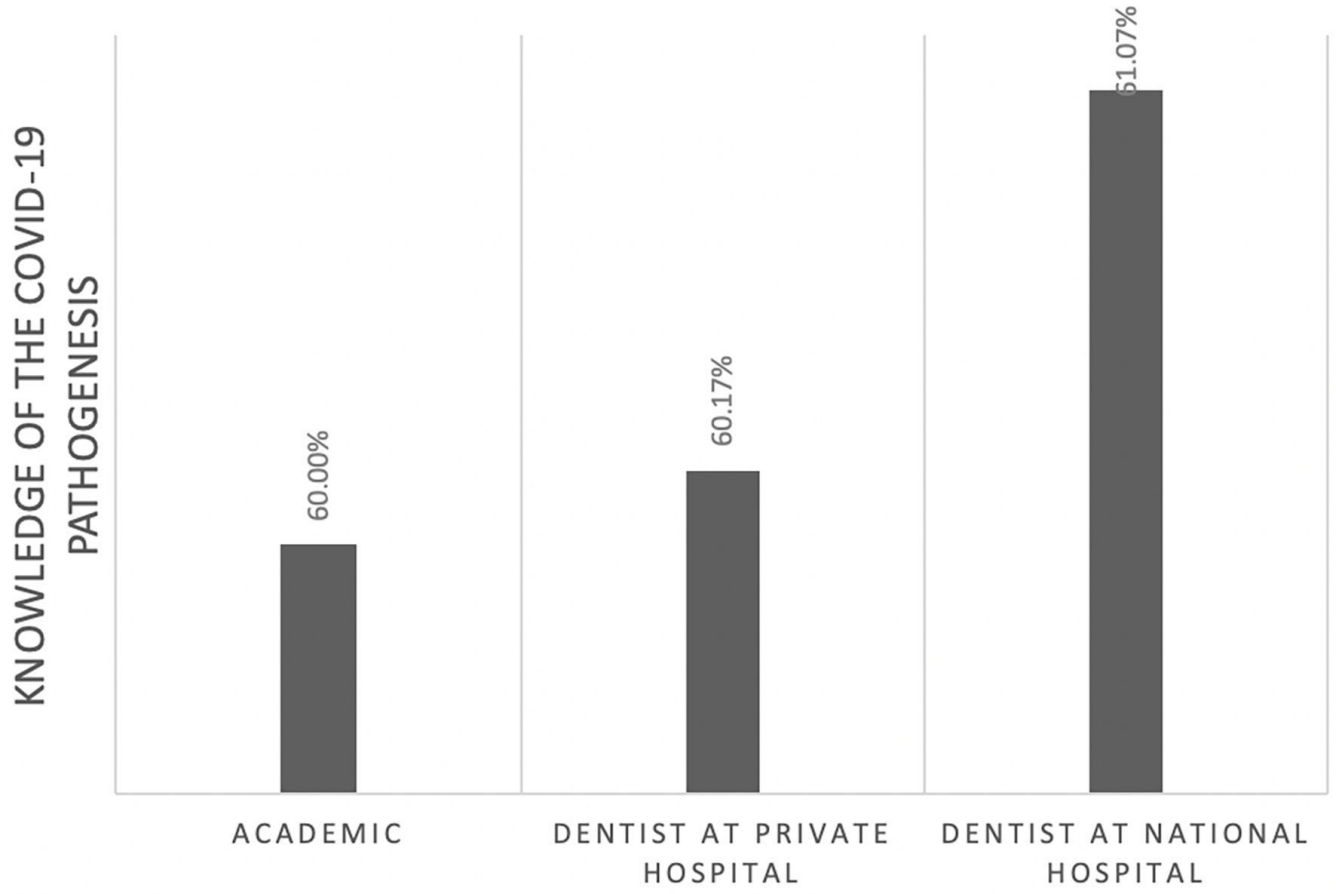
Sixty-seven percent of the participants had comprehensive knowledge about virus detection. This knowledge was not associated with the professional affiliation (*p* = 0.54).

**Figure 3:**
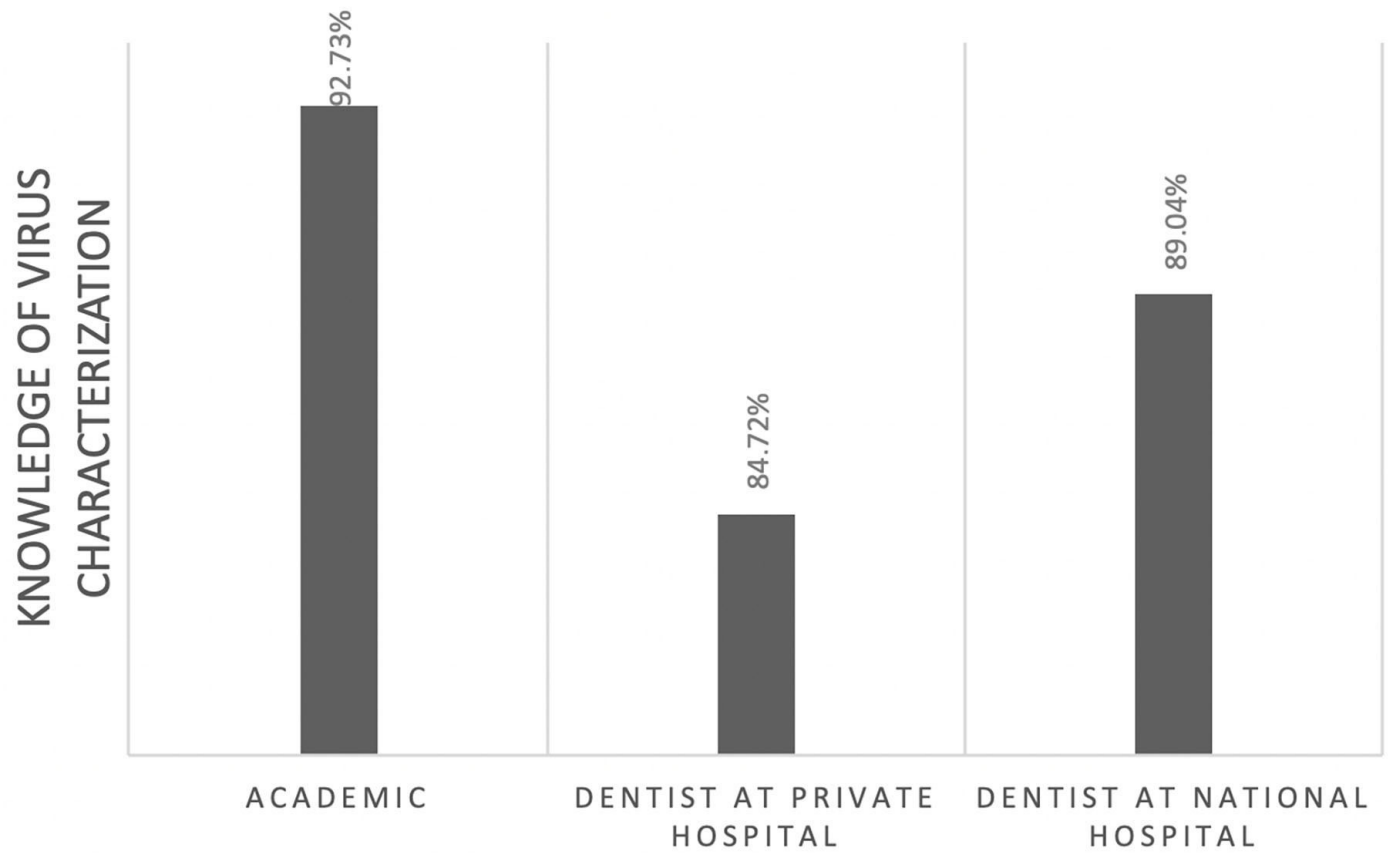
Eighty-nine percent of the participants correctly identified the characterization of the virus. This knowledge was associated with the professional affiliation (*p* < 0.05).

**Figure 4:**
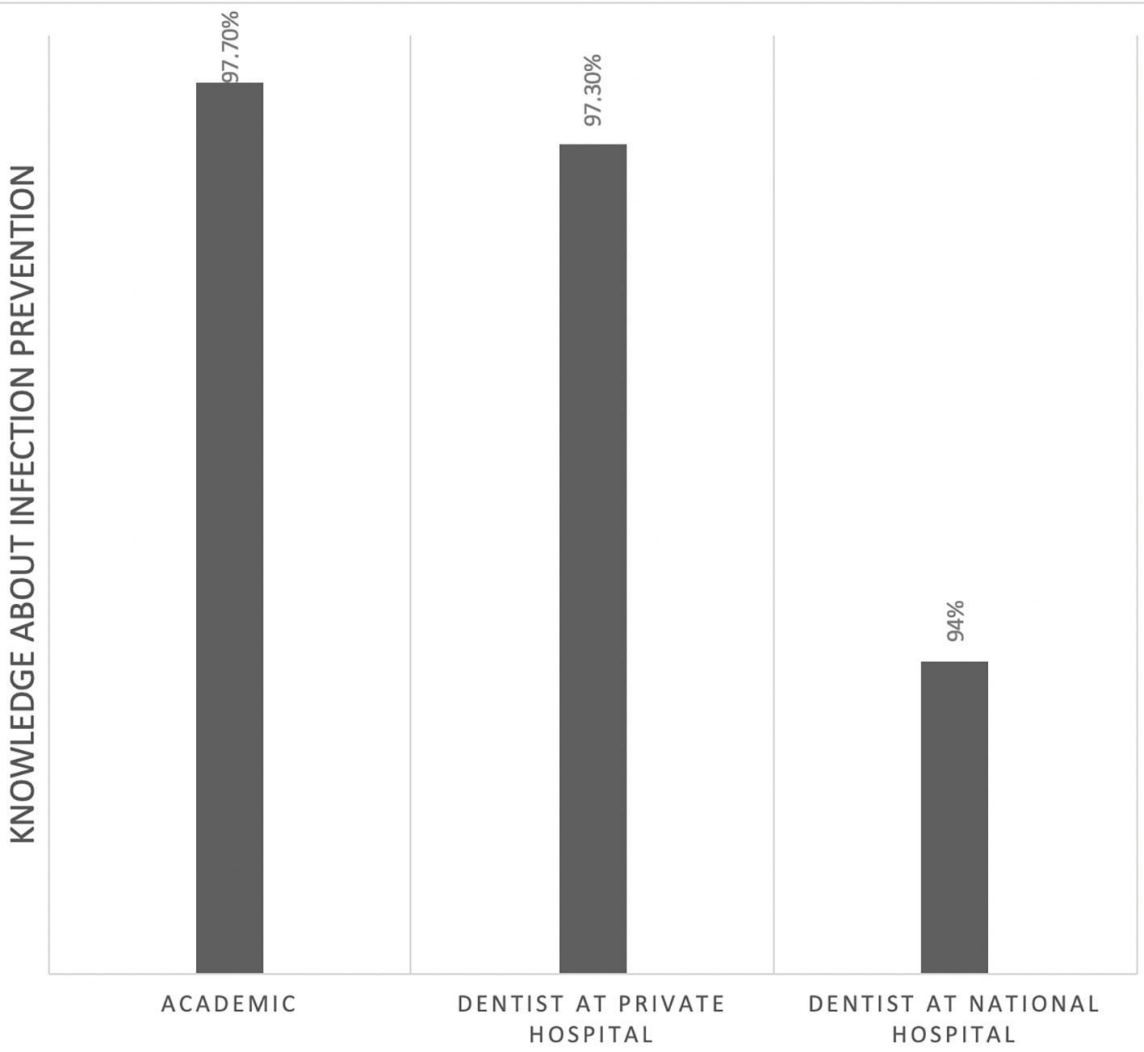
Ninety-six percent of the participants were knowledgeable about infection prevention methods. The distribution showed a significant association between professional affiliation and knowledge (*p* < 0.05).

**Figure 5:**
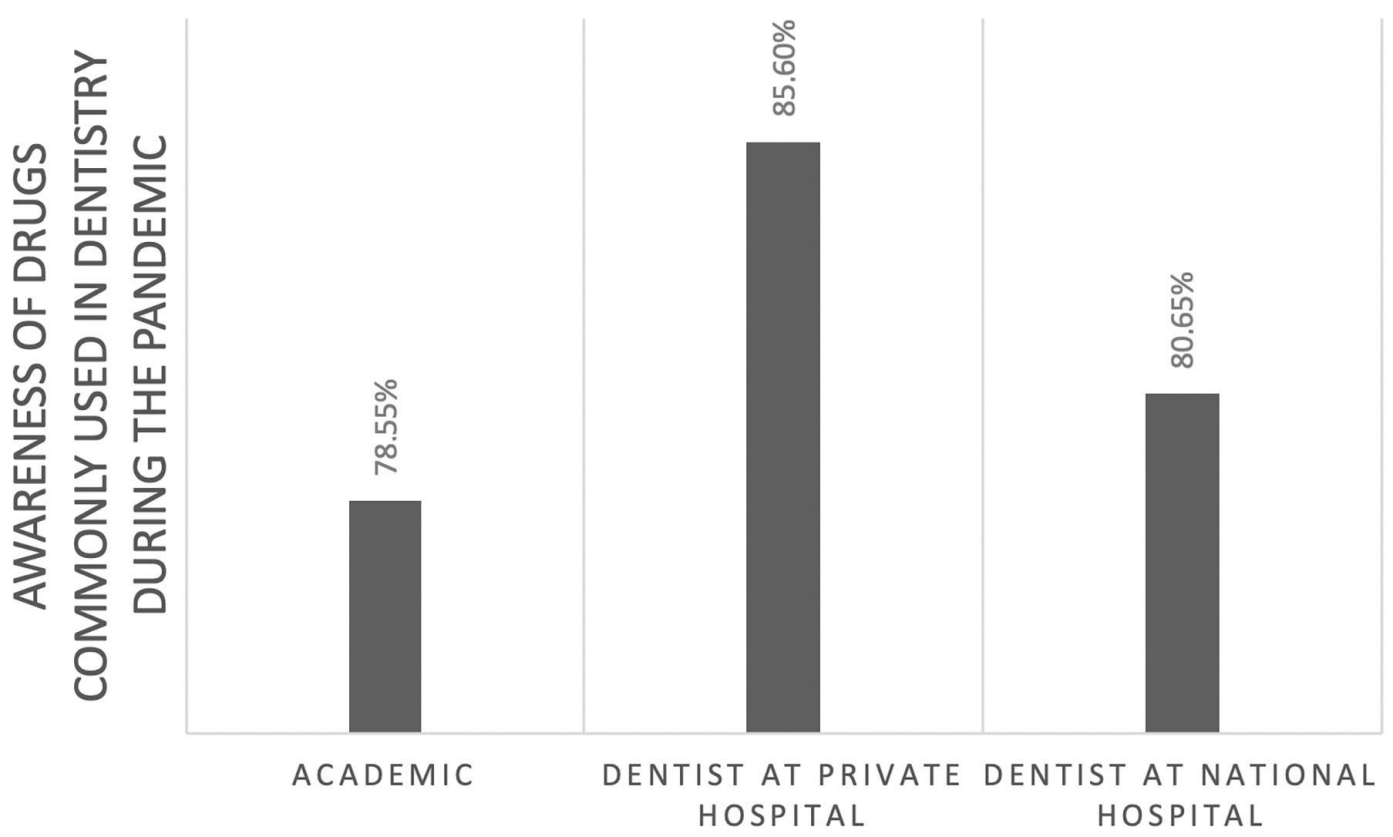
Eighty-two percent of the participants correctly identified the drugs of choice. The distribution showed a significant association between professional affiliation and knowledge (*p* < 0.05).

**Figure 6:**
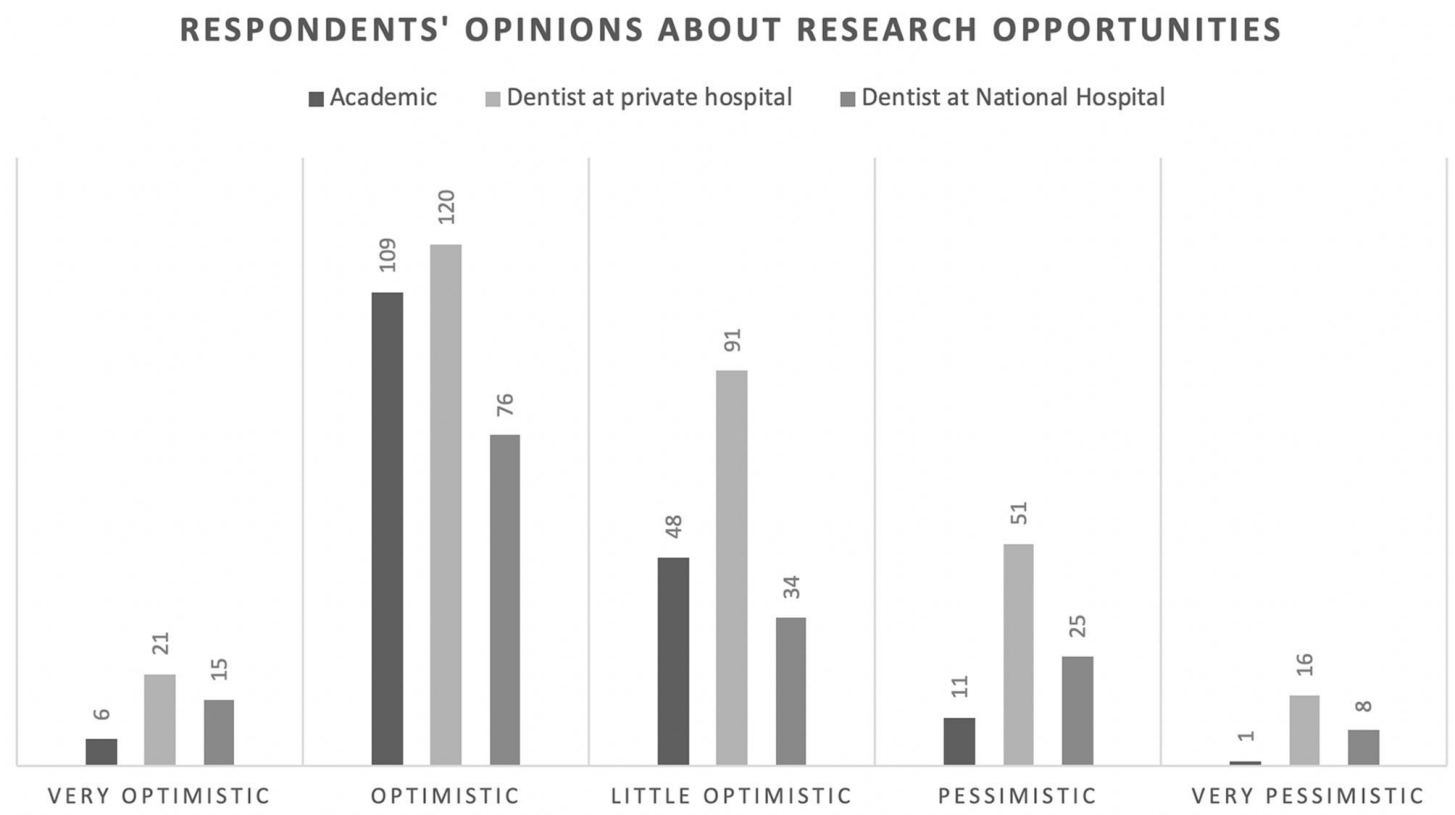
All respondents were optimistic regarding research opportunities (*p* < 0.01).

**Figure 7:**
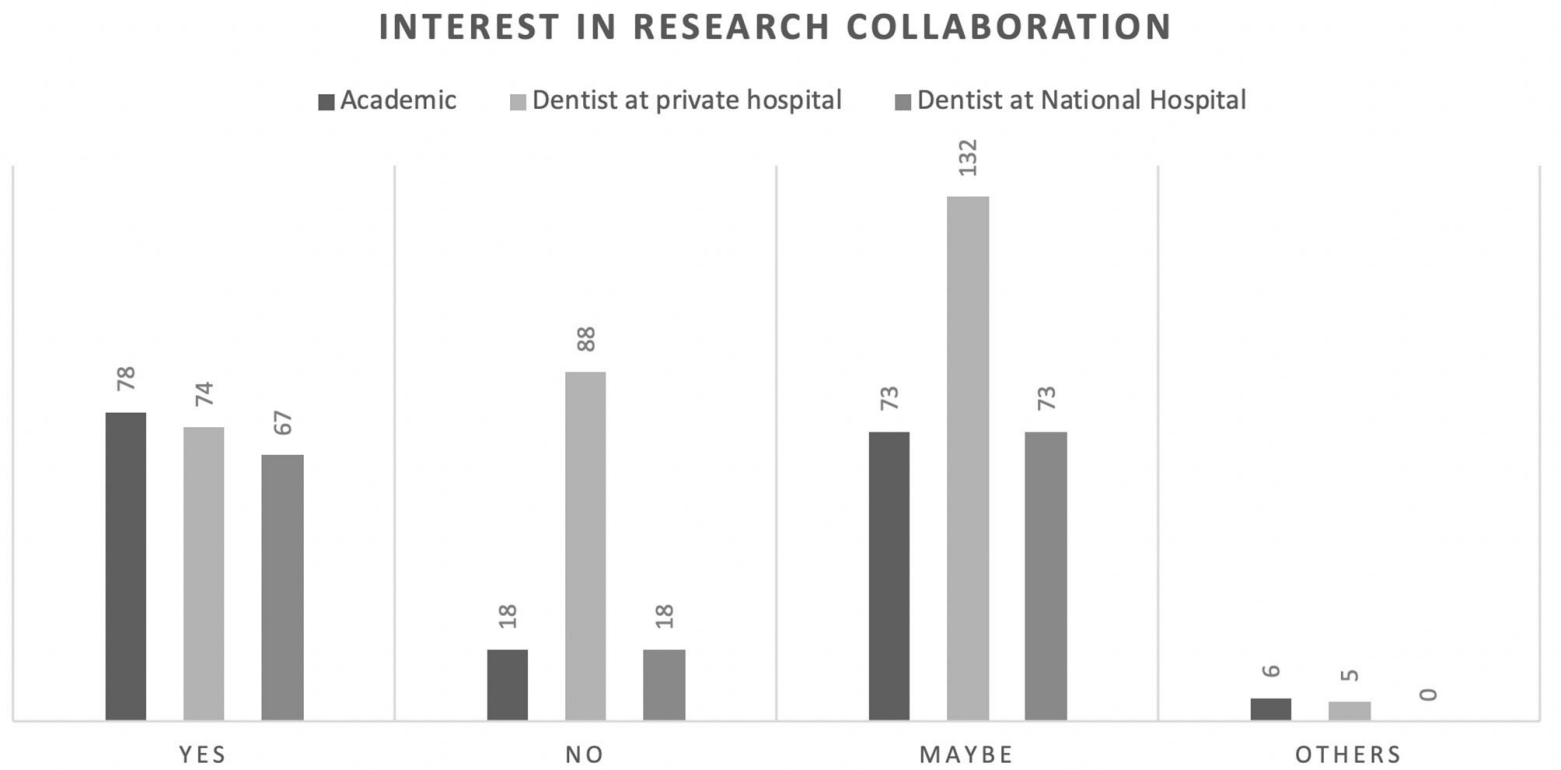
Respondents from dental faculties were more interested in research collaboration with governmental institutions (*p* < 0.01).

Of those, 60% correctly identified the pathogenesis of COVID-19 (Figure 1). However, this knowledge was not significantly associated with their professional affiliation (*p* = 0.95). Sixty-seven percent had comprehensive knowledge about virus detection methods (Figure 2). This knowledge was not associated with their professional affiliation either (*p* = 0.54). Questions regarding virus characterization, prevention and drugs of choice for the dental treatment during COVID-19 pandemic were correctly answered by 89, 96, and 82% of the participants, respectively (Figures 3–5). Knowledge related to these aspects was significantly associated with the professional affiliation (*p* < 0.05). All respondents were optimistic regarding research opportunities (*p* < 0.01) (Figure 6). Respondents with an academic background from the dental schools were more interested in participating in collaborative research with governmental institutions (*p* < 0.01) (Figure 7).

## Discussion

This is the first study to assess the knowledge of COVID-19 among the dental professional in Indonesia. The most of the participants (47.3%) were dentists practicing in private hospitals or private practitioners. This result may be attributed to the fact that the recent increase of number of private dental hospitals in Indonesia. Although the government has launched an infrastructure development strategy in the recent years, with a target of 54 government dental hospitals in 2018 and 64 hospitals in 2019, much of this growth were seen in the private healthcare sector (8). Hence, it is important to educate the private dental practitioners in Indonesia on the post-COVID infection control measures.

The role of dental professionals in preventing the transmission of COVID-19 is critically important. In this study, 96% of the participants were knowledgeable about infection prevention methods. The distribution showed a significant association between professional affiliation and knowledge. In a similar study in Jordan which conducted among practicing dentists, it was found that 71.7% were aware of mode of transmission of COVID-19, and infection controls measures in dental clinics, and has the perspective that COVID-19 is a dangerous disease (9).

Our results showed that the knowledge regarding the general information and transmission, of COVID-19 among the dentist in Indonesia is sufficient, since most of dentists answer the survey questions correctly. Not only that, they also acknowledge their profession as a high-risk zone for virus transmission. This knowledge was associated with the professional affiliation, as dentists affiliated in private hospital and academics had a better knowledge compared to dentists in national hospital (*p* < 0.05). However, the majority of dentist who participated in this study was not able to differentiate the clinical symptom between common cold and COVID-19, as both of them share the same symptoms, such as fever, cough, sore throat, headache, and fatigue. Moreover, COVID-19 patients could be asymptomatic or even without fever. Therefore, suspected cases should be tested positive with specific molecular tests to confirm (10). Similar observations have been reported by researchers in United Arab Emirates, Iran and India in a study led by Bhagavathula et al. involving health care workers globally showed that factors such as age and profession were associated with inadequate knowledge and a poor perception of COVID-19 (11). Another study conducted in Saudi Arabia showed that the basic knowledge on COVID-19 among the dental health care workers in Saudi Arabia is acceptable (12).

Herein we also assessed how professional background and affiliation affect the knowledge of COVID-19 among Indonesian dentists. In general, dentists irrespective of their professional background i.e., in private practice or academics in dental school had a good understanding regarding SARS CoV-2, COVID-19 and its relationship with their professional affiliation, and the differences between the virus and the disease. However, our data showed that academics were more knowledgeable about the characterization of SARS-CoV-2, whereas private hospital dentists tended to know more about the identification method of SARS-CoV-2. This may be due to specific professional interests. Dentists in an academic background especially affiliated to basic sciences are likely to be more interested in biomolecular and technological approaches, while practicing dentists are more interested in applied sciences (13).

Private hospital dentists showed a better understanding of medicines used in dentistry than the other two groups. Academics exhibited the lowest level of understanding. The academics teaching basic science subjects in the dental schools may not engage in clinical practice which may have contributed to their relatively lower understanding of the medicine. On the contrary, private hospital dentists who engage in patient care on daily basis continuously learn about developments in medicines to uphold their professional care (14).

At the beginning of the COVID-19 pandemic, a study claimed that taking ibuprofen could increase the risk factor of severe with SARS-CoV-2 because it could increase the levels of angiotensin-converting enzyme 2, to which the virus binds to infect the host (15). Subsequently, studies reported that ibuprofen was not found to increase risk of severe consequences from SARS CoV-2 (16–18). This is an example of why practicing dentists follow the latest developments about safe analgesics during the pandemic, as shown by the results of this study.

Dentists with academic background had a better understanding of infection control measures than the other two groups. Comparatively dentists serving government hospitals had a lowest understanding of infection control. Knowledge of infection prevention and daily preventive practices is critical during the COVID-19 pandemic.

Academics were the most optimistic regarding research opportunities (65.7%). Moreover, they were more willing to collaborate with governmental institutions (41.1%) in COVID-19 research. This is in line with the increasing number of studies on COVID-19. As of the time of writing this manuscript, according to the WHO, 39,608 COVID-19-related studies were published globally (19). Despite this exponential growth of research on COVID-19, further studies are needed to address many remaining research questions.

## Conclusion

Although knowledge and awareness of COVID-19 among Indonesian dentists are reasonably good, further improvement would be beneficial to manage patients during this pandemic. As the number of COVID-19 cases continue to rise, it is important that dentists keep abreast of the updated knowledge on this moving field. The Indonesian government has assumed a major role in containing the spread of COVID-19, however more efforts are needed to protect dentists in their daily practice. Their knowledge on infection control should be strengthened through continuous educational programs.

## Data Availability Statement

The raw data supporting the conclusions of this article will be made available by the authors, without undue reservation.

## Ethics Statement

The studies involving human participants were reviewed and approved by Institutional Review Board of Faculty of Dentistry, Trisakti University (Jakarta, Indonesia). The patients/participants provided their written informed consent to participate in this study.

## Author Contributions

ASW, EWB, and BMB contributed to the concept and design of the study. ASW and CFT collected and analyzed the data. ASW, MSD, and DNS interpreted the results. CFT, MIR, ASW, and MOR wrote sections of the manuscript. All authors contributed to the article and approved the submitted version.

## Conflict of Interest

The authors declare that the research was conducted in the absence of any commercial or financial relationships that could be construed as a potential conflict of interest.
