# Supplementary material for: COVID-19 Awareness Among Dental Professionals in Indonesia
Source: Front Med (Lausanne). 2020 Nov 4;7:589759. doi: 10.3389/fmed.2020.589759 (PMC7672024; doi:10.3389/fmed.2020.589759)
Supplement: Supplementary file 1 [file Data_Sheet_1.docx]

**Questionnaires**

1. **Knowledge of the COVID-19 pathogenesis**
   1. SARS-Cov-2 bind to Angiotensin Converting Enzyme 2 (ACE2) receptor to enter the host target cells
   2. Angiotensin Converting Enzyme 2 (ACE2) is widely distributed in alveolus/heart/colon/oral mucosa/skin .
2. **Knowledge about virus detection**
   1. Diagnosis and Virus detection using RNA methodology should be done by Real Time PCR & Aptamer
   2. Detection of SARS-Cov-2 should be conducted in biosafety level 2 laboratory and detection of the origin or mutation of virus genome should be done by genome sequencing
3. **Knowledge of virus characterization**
   1. About the disease : could name the disease correctly (**COVID-19**/SARS-Cov-2/Coronavirus) and about the virus : could name the virus correctly (COVID-19/Pneumonia/ **SARS-Cov-2**)
   2. Coronavirus is very contagious and this virus can be spread through splatter, droplet and aerosol. Dental practice is one of high risk zone for virus transmission.
   3. Coronavirus can be found in saliva and dentists have greatest risk of virus transmission
4. **Knowledge about infection prevention**
5. Dentist took preventive measures against COVID-19 included the patients travel history in their medical record and sensitized as per the WHO guidelines.
6. Maintain their health by improving their immune system through healthy life style, mouth rinse regularly and consume Indonesian herbal such as “Temulawak-Kunyit-Jahe”
7. **Awareness of drugs commonly used in dentistry during the pandemic**
   1. Drug of choice in dentistry during pandemic situation COVID-19 treatment (NSAIDs)
   2. Using drug of choice that appropriate for toothache treatment, safe and have no contraindication for patient with COVID-19 such as tramadol/paracetamol/diclofenac sodium
8. **Respondents' opinions about research opportunities and Interest in research collaboration**
   1. How optimistic to do study on this pandemic situations?

(very optimictic/optimistic/less optimistic/pessimistic/very pessimistic)

- 1. Willingness to do COVID-19 research collaboration with government

(Yes/no/maybe)
